# Towards an Accurate MRI Acute Ischemic Stroke Lesion Segmentation Based on Bioheat Equation and U-Net Model

**DOI:** 10.1155/2022/5529726

**Published:** 2022-07-16

**Authors:** Abdelmajid Bousselham, Omar Bouattane, Mohamed Youssfi, Abdelhadi Raihani

**Affiliations:** Laboratory SSDIA, ENSET Mohammedia, University Hassan 2 Casablanca, Morocco

## Abstract

Acute ischemic stroke represents a cerebrovascular disease, for which it is practical, albeit challenging to segment and differentiate infarct core from salvageable penumbra brain tissue. Ischemic stroke causes the variation of cerebral blood flow and heat generation due to metabolism. Therefore, the temperature is modified in the ischemic stroke region. In this paper, we incorporate acute ischemic stroke temperature profile to reinforce segmentation accuracy in MRI. Pennes bioheat equation was used to generate brain thermal images that may provide rich information regarding the temperature change in acute ischemic stroke lesions. The thermal images were generated by calculating the temperature of the brain with acute ischemic stroke. Then, U-Net was used in this paper for the segmentation of acute ischemic stroke. A dataset of 3192 images was created to train U-Net using *k*-fold crossvalidation. The training time was about 10 hours and 35 minutes in NVIDIA GPU. Next, the obtained trained model was compared with recent methods to analyze the effect of the ischemic stroke temperature profile in segmentation. The obtained results show that significant parts of acute ischemic stroke and background areas are segmented only in thermal images, which proves the importance of using thermal information to improve the segmentation outcomes in MRI diagnosis.

## 1. Introduction

Ischemic stroke lesion is a neurovascular abnormality caused by a sudden reduction of blood flow in some regions of the brain due to an artery occlusion, which can cause the death of cerebral tissue. It represents the commonest type of stroke, accounting for approximately 80% of stroke cases [1], and represents one of the most common reasons for death and disability globally [2, 3]. Depending on the time passed since onset, ischemic stroke can be partitioned into three phases: acute (between 0 and 24 h), subacute (between 24 h and 2 weeks), and chronic (more than 2 weeks) [2]. In the acute phase, ischemic stroke tissue can be separated into three different areas based on the potential of tissue solvability, which are infarct core, penumbra, and benign oligemia. Oligemia is a tissue slightly hypoperfused but is not at risk of death, penumbra is a tissue that is hypoperfused leading to cell death but can be recovered if the perfusion is restored rapidly, otherwise, will be destined for infarction, and infarct core is an irreversible tissue which dies as a consequence of ischemic stroke. The structure of ischemic stroke in the acute phase is concentric, where the infarct core is located at the center surrounded by penumbra, and oligemia is located in the outer area [4].

The segmentation of acute ischemic stroke lesions from MRI images is a complex and challenging problem, as its appearance changes significantly over time. Ischemic stroke lesions may not appear as homogeneous areas. Furthermore, they can be located in different regions of the brain and take different shapes [2]. Hence, effective acute ischemic stroke lesion segmentation needs the combination of different MRI modalities, as the lesion regions have diverse appearances depending on the imaging modality [5]. Each MRI modality provides different biological information, and combining different modalities provide additional informative data to have more accurate lesion borders delineation, especially in the lesion boundaries where it is challenging to differentiate between normal and abnormal pixels. In this work, we attempt to use brain temperature distribution to analyze the effect of temperature variations in acute ischemic stroke region in segmentation towards creating a new MRI modality named thermal image, which can be combined with the existing modalities for more effective detection and segmentation of acute ischemic stroke lesions.

Since manual segmentation of acute ischemic stroke is a complex and time-consuming task, which takes on average time about 15 minutes per case [2], automatic methods are of high interest for acute ischemic stroke segmentation from MRI scans. In 2015, Ischemic Stroke Lesion Segmentation (ISLES) challenge was organized by the Medical Image Computing and Computer-Assisted Intervention (MICCAI) conference [2], which provides a platform to develop and compare methods for ischemic stroke lesion segmentation from MRI scans. The first edition included penumbra estimation in acute ischemic stroke (SPES) and subacute ischemic stroke lesion segmentation (SISS). McKinley et al. [6] developed a fully automatic method based on a modified random forest algorithm, image texture, and spatial features for segmenting the ischemic penumbra. Maier et al. [7] created a segmentation model using random forest classifier combined with voxel-based hand-crafted features, and the model received high ranks in ISLES 2015 and BRATS 2015 challenges. Deep learning automated algorithms mainly convolutional neural networks (CNN) [8] were increasingly used over the recent years for acute ischemic stroke segmentation, due to the performance of deep convolutional neural networks algorithms developed in several computer vision tasks, which performed better than classical methods. CNN models represent successive convolutional layers that convolve trainable filters with the input image to extract complex features with minimal preprocessing.

In recent years, several models based on fully convolutional neural networks have been developed in the literature for ischemic stroke segmentation. Clèrigues et al. [9] proposed a deep learning network based on U-Net [10] designed for automatic acute/subacute ischemic stroke segmentation from multimodal MRI. U-Net [10] is widely used for medical image segmentation and becomes state of the art for stroke segmentation [9]. Liu et al. [11] proposed a deep learning architecture called Res-CNN for automatic acute ischemic stroke segmentation from multiple MRI modalities, and they obtained a dice score of 88.43 in SPES challenge by integrating a residual unit into a similar U-shape network. Abulnaga and Rubin [12] presented a fully CNN to segment ischemic stroke lesions from CT perfusion images, and the proposed architecture is inspired by PSPNet, which is a model that employs pyramid pooling that makes region-based context aggregation. The proposed network has been trained using focal loss function to learn more complex ischemic stroke lesion shapes. Karthik et al. [13] proposed an architecture that correlates the context from multiscaled feature maps for better segmentation of ischemic lesions. The proposed architecture uses classification and segmentation functional heads to counter class imbalance problem. It was evaluated on the ISLES 2015 SISS dataset and achieved a mean dice coefficient of 0.775. Zhang et al. [14] presented a review paper about recent deep learning methods developed for ischemic stroke lesion segmentation. In the present paper, we used U-Net architecture for acute ischemic stroke penumbra estimation from temperature distribution, and then the results were analyzed and compared with recently developed algorithms to analyze the effect of temperature on acute ischemic stroke segmentation.

The distribution of temperature in acute ischemic stroke lesion area is altered as a consequence of reduced cerebral blood flow [4]. Experimental measurement of brain temperature is limited by some issues, such as safety and ethical considerations in invasive procedures, and low accuracy of noninvasive procedures [4], such as proton magnetic resonance spectroscopy (MRS). In this work, we used Pennes bioheat equation that may provide rich information about temperature distribution changes in acute ischemic stroke lesions. Several studies have applied Pennes equation for modeling heat transfer in acute ischemic stroke lesions, by reducing cerebral blood flow and metabolic heat production. Konstas et al. [15] simulated temperature distribution in acute ischemic stroke by reducing blood perfusion to 40% and metabolic heat output to 50% of their baseline values in the ischemic penumbra and reduced blood perfusion and metabolic heat output to 25% and 30% of their baseline values in infarct core. Lillicrap et al. [4] reduced blood perfusion to 80% of its baseline value in oligemia, 40% of its baseline value in Penumbra, and 20% of its baseline value in infarct core. Due to the limited data on metabolic heat generation, they simulated three scenarios to consider different potential possibilities. Lillicrap et al. [4] performed a comparative study between simulated temperature using Pennes bioheat equation and in vivo data. The obtained results showed that Pennes bioheat equation provides temperature data corresponding to in vivo temperature for normal brain but did not correspond to temperature data available for acute ischemic stroke lesion tissue, especially in the penumbra, which is of high interest in diagnosis as it can be salvaged by reperfusion. In this paper, we still use Pennes equation, as our focus is the way the temperature is changed, and how the temperature is changed in the lesion. In a recent work [16], we proved that considering different temperature profiles of the same lesion, the obtained segmentation still accurate, which is the reason in this work we used Pennes bioheat equation.

In this paper, we exploit thermal information for acute ischemic stroke segmentation using simulated thermal images. In a recent paper [17], we segmented brain ischemic stroke lesions from temperature distribution. The ischemic stroke lesion segmentation was carried out using U-Net neural network based on temperature changes in the lesion zone. The temperature distribution in the brain with the ischemic stroke was calculated using the Pennes equation. Then, U-Net was used for ischemic stroke segmentation from the generated thermal images. However, we considered just a small dataset of 440 thermal images and 19 thermal images to test the model. In this present paper, we used 3192 thermal images with additional noise for training the U-Net based fully CNN, which showed impressive results in medical image analysis [18]. Furthermore, we use a modified version of Pennes equation by considering a heterogeneous thermal conductivity for more accurate temperature calculation.

## 2. Methodology

### 2.1. Thermal Image Calculation

The heat transfer in a biological system is modeled using Pennes bioheat transfer equation [19], which provides simplifying assumptions about vascular architecture, blood perfusion, equilibration site, and temperature of blood [20]. Accordingly, several other models tried to overcome these limitations by considering more complex formulations [21–24]. Due to simplicity, effectiveness, and ease of use, Pennes equation stills commonly used in the literature for the calculation of temperature distribution and interpretation of thermal data. Pennes equation describes the effects of metabolic heat production and blood flow on the heat energy equilibrium in biological tissue. These two effects were added to the standard heat equation and were written in the following form:

(1) $$\begin{array}{c} \rho C_{P}\frac{\partial T}{\partial t}=\nabla \left(\kappa \nabla \text{T}\right)+\omega_{b}\rho_{b}C_{pb}\left(T_{a}-T\right)+Q_{m}, \end{array}$$

where *ρ*[kg/m^3^] is the density of the tissue, *C*_*P*_[J/kg°C] is the specific heat of the tissue, *κ*[W/m°C] is the thermal conductivity, *ω*_*b*[ml/s.ml]_ is the blood perfusion rate, *ρ*_*b*_[kg/m^3^] is the density of the blood, *C*_*pb*_[J/kg°C] is the specific heat of the blood, *T*_*a*_[°C] is the temperature of the artery, and *Q*_*m*_[W/m^3^] is the metabolic heat generation.

To solve Eq. (1), we considered the normal body temperature *T*_*i*_ = 37°C as an initial condition and 37°C as a boundary condition. The blood temperature was set to *T*_*a*_ = 36.7°C [25], and the remaining blood perfusion thermal properties have been obtained from our recent paper [16]. Since Pennes equation is a modified version of the standard heat diffusion energy equation, it can be discretized based on numerical methods, such as finite volume method (FVM) [26], lattice Boltzmann method [27], and finite difference method (FDM) [28]. In this work, we used FDM to solve the Pennes bioheat equation numerically in a two-dimensional Cartesian grid. We consider Δ*t* = 0.1s as a time step and Δ*x* = Δ*y* = 2 mm as a spatial step (the same as the spatial resolution of SPES images). The convergence of the FDM solver is reached when the temperature difference at all nodes between two successive iterations is less than 1 × 10^−7^. The process of discretization is detailed in [29], and the obtained discretized form is presented as follows:

(2) $$\begin{array}{c} \;T_{i,j}^{n+1}=T_{i,j}^{n}+\frac{∆t}{\rho_{i,j}{\left(C_{P}\right)}_{i,j}∆x^{2}\;}.\left[\begin{array}{c} k_{i+1/2,j}\left(T_{i+1,j}^{n}-T_{i,j}^{n}\right)-k_{i-1/2,j}\left(T_{i,j}^{n}-T_{i-1,j}^{n}\right)\\ +k_{i,j+1/2}\left(T_{i,j+1}^{n}-{\text{T}}_{i,j}^{n}\right)-k_{i,j-1/2}\left(T_{i,j}^{n}-T_{i,j-1}^{n}\right) \end{array}\right]+\frac{∆t}{\rho_{i,j}C_{i,j}}\;\left[{\left(\omega_{b}\right)}_{i,j}{\left(\rho_{b}\right)}_{i,j}{\left(C_{Pb}\right)}_{i,j}\left(T_{a}^{n}-T_{i,j}^{n}\right)+Q_{i,j}\right], \end{array}$$

where *n* is the iteration number, *T*_*i*,*j*_^*n*^  is the temperature at (*i*, *j*) grid node, (*ω*_*b*_)_*i*,*j*_ is the blood perfusion rate at (*i*, *j*) grid node, *Q*_*i*,*j*_ is the metabolic heat generation at (*i*, *j*) grid node, *ρ*_*i*,*j*_, (*ρ*_*b*_)_*i*,*j*_ is the density of tissue and blood at (*i*, *j*) grid node, (*C*_*P*_)_*i*,*j*_, (*C*_*Pb*_)_*i*,*j*_ is the specific heat of tissue and blood at (*i*, *j*, *k*) grid node, and *k*_*i*+1/2,*j*_  represents the thermal conductivity computed at the midpoint in piecewise homogeneous media using the following formula [29]:

(3) $$\begin{array}{c} k_{i+1/2,j}=\frac{2k_{i,j}k_{i+1,j}}{k_{i,j}+k_{i+1,j}}. \end{array}$$

The thermophysical properties of normal brain tissues (GM, WM, and CSF) and ischemic stroke tissues (penumbra and infarct core) are presented in Table 1. In the present work, we analyzed heterogeneous acute ischemic stroke lesions, with two types of tissues, infarct area, which represents the region that has already infarcted and cannot be recovered, and penumbra which is a hypoperfused tissue leading to cell death but can be recovered if the perfusion is restored rapidly [4]. The thermophysical properties of infarct core and penumbra were obtained from white matter by reducing the values of blood perfusion and heat generation due to metabolism. In the ischemic penumbra, blood perfusion was reduced to 40% of its baseline value, and heat generation due to metabolism was reduced to 70% of its baseline value. For the ischemic infarct core, blood perfusion was reduced to 20% of its baseline value, and heat generation due to metabolism was set at 0 [4].

### 2.2. U-Net Architecture

**Figure 1:**
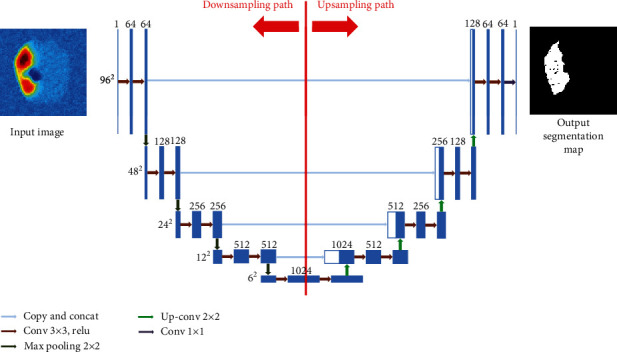
A schematic of U-Net architecture trained on thermal images. The input of the network is a normalized thermal 2D image, and the output is the segmentation map using Dice coefficient as a loss function with sigmoid activation function. The architecture includes a downsampling path and an upsampling path, with concatenation between the corresponding layers.

In this study, we used U-Net convolutional neural network for acute ischemic penumbra segmentation. This architecture was introduced by Ronneberger et al. [10] for the segmentation of biomedical images and recently showed impressive results for biomedical image segmentation [30]. The architecture of U-Net is created based on fully CNN proposed by Shelhamer et al. [31] and was changed to be trained with fewer images and modified in a way it produces precise segmentation for biomedical images.  Figure 1 shows the U-Net network used in this paper; it includes two paths: the first is the downsampling (encoding) path, and its objective is to capture the context of the input image. The second is the upsampling (decoding) path, and its purpose is to enable accurate localization using upsampling.

- The downsampling path contains 5 convolutional blocks. Each block contains two 3 × 3 convolutional layers, with a stride of 1, followed by ReLU (Rectified Linear Unit) [32] activation function. After every convolutional block, the number of feature maps is doubled, which increases from 1 to 1024, and 2 × 2 max pooling is used after every block except the last block for downsampling. Thus, the size of feature maps was reduced from 96 × 96 to 6 × 6.

(ii) The upsampling path includes a set of blocks. Every block consists of an upsampling of the feature maps with a 2 × 2 convolution, concatenated with the corresponding feature maps from the downsampling path, and then two 3 × 3 convolution layers are applied, each followed by a ReLU activation function. The last layer is a 1 × 1 convolution used to reduce the number of feature maps from 64 to 2, which represent the background and the penumbra segmentation maps

Unlike the original U-Net paper, in this work, we used zero padding to keep the input equal to the output feature maps size for all the convolutional layers. The hyperparameters used in this work for training U-Net architecture are taken from [33], and stochastic gradient descent has been used to minimize the cost function. Adaptive moment estimator (Adam) [34] was adopted for the parameter estimation with a learning rate of 0.0001. The batch size was set to 22 due to the limited GPU memory, with 100 as the maximum number of epochs. All the network weights were initialized based on a normal distribution with a mean of 0 and 0.01 of standard deviation, and all the network biases were fixed at 0. In the output layer, we used Dice coefficient described in [35] as a loss function with a sigmoid activation function. In total, the network consists of 34 layers and 31 030 593 parameters.  Table 2 shows the details of each layer where 2@Conv means that two consecutive convolution layers are applied.

### 2.3. SPES Dataset

Towards the evaluation of the proposed approach, we have used the public acute stroke penumbra estimation subtask (SPES) dataset from ISLES challenge 2015 [2]. SPES dataset contains 30 training and 20 testing cases. The images were stored as 3D volumes of 96 × 110 × 71 dimensions with isotropic spatial dimension of 2 × 2 × 2 mm. For each case, seven modalities were provided including anatomical (T1 contrast, T2), diffusion (DWI), and perfusion (CBF, CBV, TTP, Tmax) MRI.

The temperature distribution calculation using Pennes bioheat equation needs the brain tissues segmentation, to define thermal properties for each tissue, and we exploited the work of Li et al. [36] to distinguish the brain tissues from T1 MR images. The acute ischemic stroke ground truth is provided by ISLES challenge 2015 for ischemic penumbra and infarct core. The obtained segmentation of normal tissues and the provided ground truth of acute stroke lesion was used as an input to Eq. (2), which was implemented in *C* language. We created a 2D array for each thermal property of a size of 96 × 110, where each array element corresponds to an image pixel. The result of solving Eq. (2) is a 2D array of temperature, where each element of the array represents the temperature of the corresponding pixel, and then was exported in a Mat file. The temperature data was loaded in MATLAB and exported to 2D grayscale images that were used for U-Net architecture training. To keep the balance on the number of image slices for each patient, we selected 19 2D images from each patient; all the images comprise an ischemic penumbra inside. A total of 532 grayscale thermal images were created for training using *K*-fold crossvalidation, taken from the first 28 patients. Additional 38 grayscale thermal images were created from patients 29 and 30 to compare the trained network with other models from the literature. We normalized the intensity of grayscale thermal images between 0 and 1, and then the images were cropped to 96 × 96 to allow U-Net to perform segmentation.

### 2.4. Data Augmentation

**Figure 2:**
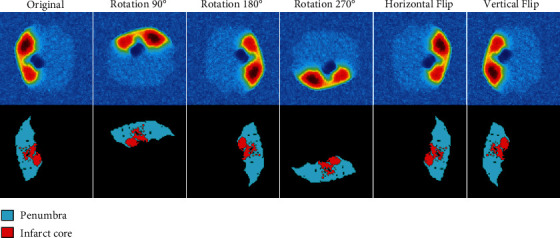
Data augmentation used to improve the network performance by increasing the size of training dataset using new images generated artificially from the existing images in the dataset. Left: the original thermal image with ground truth. The rest are rotated and flipped versions of the original thermal image.

Image data augmentation is used to improve the network performance by increasing the size of the training dataset using new images generated artificially from the existing images in the dataset. The main goal when using data augmentation is to enhance the generalizability of the model. In this paper, we performed a combination of data augmentation techniques, namely, rotation in 90°, 180°, and 270°, besides horizontal and vertical flips. The final dataset size generated for training the network is 3192 images.  Figure 2 illustrates the data augmentation results.

We used *K*-fold crossvalidation approach to train the U-Net network. Crossvalidation is a technique for evaluating machine learning models by training multiple models on subsets of the available input data and evaluating them on the complementary subset of data. In this work, we considered five folds for crossvalidation, four folds for training, and one fold for validation. We obtained five trained networks. We calculated the average of the output of the five networks in test data, and the obtained result is compared with other methods in the literature.

### 2.5. Implementation Details

The U-Net architecture has been executed using Java language based on DeepLearning4J (https://deeplearning4j.org/, version: 1.0.0-beta7) implementation, and it is an open-source deep learning framework for Java and JVM languages. The experiments have been run on a Windows 7 (64 bits, ultimate edition) operating system with a CPU Intel i7-4770k with 4 cores of 3.50 GHz, 8 threads, and 16 GB of memory. The network training and testing have been done using NVIDIA GeForce GTX 1060 (NVIDIA Corp, United States), which has 6.1 in computing capability, 1280 Cuda cores, and 6 GB in memory.

Towards the segmentation evaluation in thermal images, we used the segmentation evaluation metrics available in DeepLearning4J, which are accuracy, precision, recall, and Dice, presented in the following formulas [37]:

(4) $$\begin{array}{c} \text{Accuracy}=\frac{\left(\text{TP}+\text{TN}\right)}{\left(\text{TP}+\text{FN}+\text{TN}+\text{FP}\right)}, \end{array}$$

(5) $$\begin{array}{c} \text{Precision}=\frac{\text{TP}}{\text{TP}+\text{FP}}, \end{array}$$

(6) $$\begin{array}{c} \text{Recall}=\frac{\text{TP}}{\text{TP}+\text{FN}}, \end{array}$$

(7) $$\begin{array}{c} \text{Dice}=\frac{2*\text{TP}}{\left(2\text{TP}+\text{FP}+\text{FN}\right)}, \end{array}$$

where TP represents the true positives, FP denotes the false positives, TN indicates the true negatives, and FN signifies the false negatives.

## 3. Results

In this section, we present the temperature of the brain with acute ischemic stroke by considering two cases, simplified geometry and realistic geometry of the lesion. Next, we show the obtained results of ischemic penumbra segmentation from thermal images, and then the results are compared with recent methods taken from the literature.

**Figure 3:**
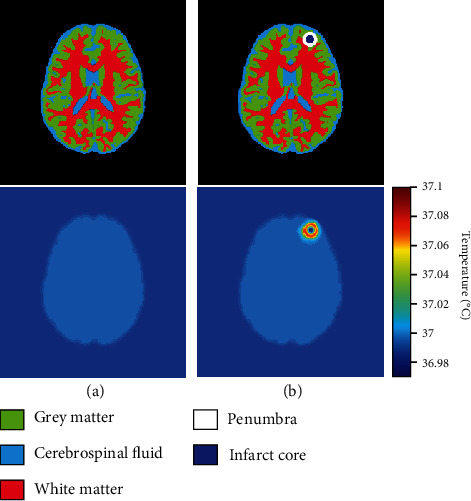
Calculated temperature distribution of brain with circular acute ischemic stroke lesion using Pennes bioheat equation. (a) Brain without acute ischemic stroke. (b) Brain with acute ischemic stroke lesion of spherical infarct core of 10 mm of diameter, surrounded by penumbra with 5 mm thick.

**Figure 4:**
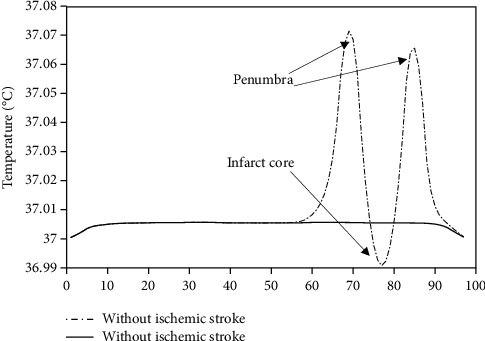
One-dimensional representation of temperature profile on the path passes through the center of the acute ischemic stroke lesion. The temperature is reduced in infarct core compared to normal tissue. However, the temperature is warmer in penumbra compared to normal brain tissue.

**Figure 5:**
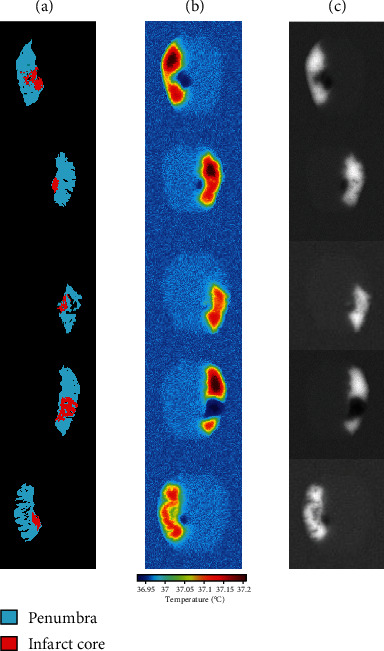
Thermal images taken from the dataset generated using Pennes bioheat equation of five patients with acute ischemic stroke lesion of different volumes and located in different regions in the brain. (a) Ground truth of acute ischemic stroke lesion. (b) Thermal images in color. (c) Grayscale thermal images.

Figures 3(a) and 3(b) illustrate the brain temperature distribution with and without acute ischemic stroke lesion, by considering a simplified geometry of acute ischemic stroke. We considered a spherical infarct core with a diameter of 10 mm, surrounded by 5 mm thick of penumbra [4].  Figure 4 illustrates a one-dimensional representation of the temperature distribution of the line passed in the lesion center. One can observe that the temperature is reduced in the infarct core compared with normal tissue; this can be explained by the reduction in cerebral blood flow and zero in metabolic heat production. However, the temperature is warmer in penumbra compared with normal brain tissue, as it is still a metabolic activity in this region.  Figure 5 shows the thermal images of five patients taken from the generated dataset; the figure contains the ground truth of penumbra merged with infarct core, the thermal images in color, and grayscale thermal images. For training and testing U-Net model, we used grayscale thermal images, obtained by transforming the temperature distribution calculated using Eq. (2) with 5% of Gaussian noise of the brain with acute ischemic stroke lesion to grayscale images.

We trained U-Net architecture in a dataset containing 3192 grayscale thermal images using *K*-fold crossvalidation approach. The evaluation of segmentation is illustrated in Table 3 using four metrics, namely, accuracy, precision, recall, and Dice. One can observe that U-Net model yields an accurate and precise ischemic stroke penumbra estimation from thermal images.

Tables 4 and 5 give the results of acute ischemic penumbra estimation in thermal images compared with Clèrigues et al. [9], Maier et al. [7], and McKinley et al. [6] methods, the segmentation results of Maier et al. [7], and McKinley et al. [6] were downloaded directly from ISLES 2015 platform [38], for Clèrigues et al. [9], and were provided by the authors of the paper. These tables illustrate the percentage of ischemic penumbra and background areas segmented in thermal images and not segmented using the other methods. The comparative study was performed in 30 2D slices taken using DeepLearning4J from subjects 29 and 30 in the SPES training dataset. For Clèrigues et al. [9], we obtained an average of 0.54% of ischemic penumbra area segmented only in thermal images and an average of 0.84% of background segmented only in thermal images. For Maier et al. [7], we obtained an average of 5.7% of ischemic penumbra area segmented only from thermal images and an average of 0.56% of background segmented only from thermal images. For McKinley et al. [6], we obtained an average of 2.71% of ischemic penumbra area segmented only in thermal images and an average of 0.81% of background segmented only from thermal images. One can observe that significant regions are segmented only from thermal images, which proves the importance of temperature information to increase the true positives and true negatives pixels and improve segmentation in conventional MRI modalities.

## 4. Discussion

The human body temperature distribution is influenced by several elements such as metabolic heat production, heat exchange processes between skin tissues, blood perfusion, circadian rhythm, and parasympathetic activity for maintaining homeostasis [39]. In the presence of abnormality like acute ischemic stroke, some of these factors change influences the distribution of temperature in the abnormality region. Therefore, the change in human body temperature can be interpreted by the presence of abnormality. The temperature has been extensively used for characterizing several abnormalities in the human body, based on changes in temperature in the abnormality area compared with surrounding tissues. Infrared thermography has been used as a screening tool to measure body surface temperature to evaluate breast tumors [40–42], skin tumors [43, 44], and eye diseases [45, 46]. It is challenging to use infrared thermography to measure the brain temperature, as it fails to measure the temperature of deep organs of the human body due to its limited depth penetration.

In previous years, different techniques were used for brain temperature measurement, invasively by inserting probes in brain tissue [47]. Invasive techniques may produce local microlesions and inflammatory responses nearby the probes, which might influence brain temperature [48]. Infrared thermal imaging (thermography) is a noninvasive technique, it has been used to measure brain temperature under the name of intraoperative thermal imaging (ITI) to delineate brain tumor borders [39, 49–51], and this technique is used during surgical resection of brain lesions. Another technique used to measure the brain temperature noninvasively is named magnetic resonance spectroscopy (MRS), which has been used to measure brain temperature in patients with brain tumors [52] and acute ischemic stroke [53]. However, MRS is limited in terms of measurement accuracy [54].

In this work, we used Pennes bioheat equation solved using FDM to calculate the brain temperature with acute ischemic stroke lesion. We created a dataset of synthetic thermal images of acute ischemic stroke that have the same resolution as other MR modalities used in the SPES dataset with an isotropic spatial dimension of 2 × 2 mm. Compared with recent methods applied in conventional MR images, we showed that some background pixels and abnormal pixels are identified only from thermal images. The obtained results can explore more studies in future works to create accurate noninvasive MR thermal scans that can be combined with other modalities such as T1c, T2, DWI, and CBF for more accurate acute ischemic stroke segmentation.

## 5. Conclusion

In conclusion, we presented in this study a method utilizing brain temperature to improve acute ischemic stroke segmentation in MRI. Acute ischemic stroke lesions modify brain temperature profiles due to the reduction of cerebral blood flow and metabolic heat production. We used Pennes bioheat equation with FDM to create a dataset of 4032 thermal images. Next, U-Net architecture was trained in the generated dataset. The trained model was compared with recent methods and showed promising results. In future works, we plan to create a tensorized version of Pennes bioheat equation using diffusion tensor imaging (DTI) to calculate more realistic brain temperature.

## Figures and Tables

**Table 1:** Thermophysical properties of the brain and acute ischemic stroke tissues.

Material	Property					
	$k\;\left[{\frac{W}{m}}^{{}^{\circ}}C\right]$	$\rho \;\left[\frac{\text{kg}}{{\text{m}}^{3}}\right]$	$C_{p}\left[{\frac{J}{\text{kg}}}^{{}^{\circ}}\text{C}\right]$	$Q_{m}\left[\frac{\text{W}}{{\text{m}}^{3}}\right]$	$\omega_{b}\left[\frac{\text{ml}}{\text{ml}}\text{s}\right]$	Refs
CSF	0.6	1000	4200	0	0	[55]
GM	0.565	1035.5	3680	16,229	0.013289	[55]
WM	0.503	1027.4	3600	4517.9	0.0036956	[55]
Penumbra	0.503	1027.4	3600	3162.53	0.00147824	[4, 55]
Infarct core	0.503	1027.4	3600	0	0.00073912	[4, 55]

**Table 2:** Detail of the architecture of U-Net.

Layers	Architectures	Output
Input	Image (96 × 96)	96 × 96 × 1
conv1	2@Conv (3 × 3)/Relu padding = ^“^same^”^	96 × 96 × 64
	Max pooling stride = 2	
conv2	2@Conv (3 × 3)/Relu padding = ^“^same^”^	48 × 48 × 128
	Max pooling stride = 2	
conv3	2@Conv (3 × 3)/Relu padding = ^“^same^”^	24 × 24 × 256
	Max pooling stride = 2	
conv4	2@Conv (3 × 3)/Relu padding = ^“^same^”^	12 × 12 × 512
drop4	Dropout (*p* = 0.5)	
	Max pooling stride = 2	
conv5	2@Conv (3 × 3)/Relu padding = ^“^same^”^	6 × 6 × 1024
drop5	Dropout (*p* = 0.5)	
up6	Upsampling conv (2 × 2)/Relu	12 × 12 × 512
Concatination	[drop4, up6]	
conv6	2@Conv (3 × 3)/Relu padding = ^“^same^”^	12 × 12 × 512
up7	Upsampling conv (2 × 2)/Relu	24 × 24 × 256
Concatination	[conv3, up7]	
conv7	2@Conv (3 × 3)/Relu padding = ^“^same^”^	24 × 24 × 256
up8	Up-sampling conv (2 × 2)/Relu	48 × 48 × 128
Concatination	[conv2, up8]	
conv8	2@Conv (3 × 3)/Relu padding = ^“^same^”^	48 × 48 × 128
up9	Upsampling conv (2 × 2)/Relu	96 × 96 × 64
Concatination	[conv1, up9]	
conv9	2@Conv (3 × 3)/Relu padding = ^“^same^”^	96 × 96 × 64
conv10	Conv (1 × 1) Sigmoid	96 × 96 × 1
Output	Segmentation map	96 × 96 × 1

**Table 3:** The segmentation evaluation metrics for U-Net segmentation in thermal images.

	Accuracy	Precision	Recall	Dice
Penumbra	0.99 ± 0.0004	0.92 ± 0.005	0.95 ± 0.006	0.93 ± 0.002

**Table 4:** The percentage of ischemic penumbra and background areas segmented in thermal images and not segmented using methods from the literature in slices taken from subject 29.

Slice No.	Method	Ischemic penumbra area segmented only in thermal images (%)	Background area segmented only in thermal images (%)
Slice 31	Clèrigues et al. [9]	0.0	0.25
	Maier et al. [7]	2.25	0.36
	McKinley et al. [6]	0.61	0.21
Slice 32	Clèrigues et al. [9]	3.47	0.46
	Maier et al. [7]	5.31	0.31
	McKinley et al. [6]	6.41	0.17
Slice 33	Clèrigues et al. [9]	0.0	0.82
	Maier et al. [7]	2.06	0.17
	McKinley et al. [6]	2.62	0.11
Slice 34	Clèrigues et al. [9]	0.0	0.78
	Maier et al. [7]	3.87	0.06
	McKinley et al. [6]	2.64	0.09
Slice 35	Clèrigues et al. [9]	0.0	0.95
	Maier et al. [7]	2.66	0.06
	McKinley et al. [6]	3.16	0.15
Slice 36	Clèrigues et al. [9]	0.0	1.28
	Maier et al. [7]	2.34	0.05
	McKinley et al. [6]	0.67	0.11
Slice 37	Clèrigues et al. [9]	0.0	1.0
	Maier et al. [7]	2.49	0.0
	McKinley et al. [6]	1.87	0.05
Slice 38	Clèrigues et al. [9]	0.31	0.65
	Maier et al. [7]	4.97	0.02
	McKinley et al. [6]	3.26	0.03
Slice 39	Clèrigues et al. [9]	0.0	0.46
	Maier et al. [7]	7.81	0.08
	McKinley et al. [6]	4.9	0.04
Slice 40	Clèrigues et al. [9]	2.0	0.59
	Maier et al. [7]	10.8	0.02
	McKinley et al. [6]	6.83	0.01
Slice 41	Clèrigues et al. [9]	0.63	0.71
	Maier et al. [7]	9.74	0.1
	McKinley et al. [6]	6.7	0.08
Slice 42	Clèrigues et al. [9]	0.16	0.79
	Maier et al. [7]	8.66	0.03
	McKinley et al. [6]	3.69	0.08
Slice 43	Clèrigues et al. [9]	0.96	1.0
	Maier et al. [7]	10.33	0.03
	McKinley et al. [6]	3.23	0.04
Slice 44	Clèrigues et al. [9]	1.73	0.82
	Maier et al. [7]	12.8	0.01
	McKinley et al. [6]	4.84	0.08
Slice 45	Clèrigues et al. [9]	1.04	0.51
	Maier et al. [7]	10.0	0.0
	McKinley et al. [6]	2.91	0.06
Average	Clèrigues et al. [9]	0.69	0.74
	Maier et al. [7]	6.41	0.09
	McKinley et al. [6]	3.62	0.09

**Table 5:** The percentage of ischemic penumbra and background areas segmented in thermal images and not segmented using methods from the literature in slices taken from subject 30.

Slice No.	Method	Ischemic penumbra area segmented only in thermal images (%)	Background area segmented only in thermal images (%)
Slice 27	Clèrigues et al. [9]	2.29	0.86
	Maier et al. [7]	3.21	1.0
	McKinley et al. [6]	1.6	0.63
Slice 28	Clèrigues et al. [9]	0.0	1.22
	Maier et al. [7]	0.23	1.02
	McKinley et al. [6]	0.0	0.72
Slice 29	Clèrigues et al. [9]	0.45	1.37
	Maier et al. [7]	0.67	1.0
	McKinley et al. [6]	1.12	0.98
Slice 30	Clèrigues et al. [9]	0.61	0.93
	Maier et al. [7]	7.56	1.19
	McKinley et al. [6]	2.24	0.79
Slice 31	Clèrigues et al. [9]	0.40	0.92
	Maier et al. [7]	14.83	1.31
	McKinley et al. [6]	4.47	0.87
Slice 32	Clèrigues et al. [9]	0.19	0.96
	Maier et al. [7]	15.29	1.19
	McKinley et al. [6]	4.58	0.85
Slice 33	Clèrigues et al. [9]	1.34	0.97
	Maier et al. [7]	10.74	1.23
	McKinley et al. [6]	3.07	0.82
Slice 34	Clèrigues et al. [9]	0.35	0.78
	Maier et al. [7]	6.57	1.05
	McKinley et al. [6]	2.66	0.57
Slice 35	Clèrigues et al. [9]	0.17	0.92
	Maier et al. [7]	2.38	1.01
	McKinley et al. [6]	1.02	0.44
Slice 36	Clèrigues et al. [9]	0.15	0.56
	Maier et al. [7]	1.68	0.78
	McKinley et al. [6]	0.92	0.42
Slice 37	Clèrigues et al. [9]	0.14	0.54
	Maier et al. [7]	1.56	0.56
	McKinley et al. [6]	0.56	0.37
Slice 38	Clèrigues et al. [9]	0.0	0.86
	Maier et al. [7]	1.41	0.74
	McKinley et al. [6]	1.13	0.58
Slice 39	Clèrigues et al. [9]	0.0	0.88
	Maier et al. [7]	2.56	0.83
	McKinley et al. [6]	0.99	0.68
Slice 40	Clèrigues et al. [9]	0.0	1.13
	Maier et al. [7]	3.02	1.19
	McKinley et al. [6]	1.05	0.97
Slice 41	Clèrigues et al. [9]	0.0	1.16
	Maier et al. [7]	2.98	1.31
	McKinley et al. [6]	1.56	1.09
Average	Clèrigues et al. [9]	0.40	0.94
	Maier et al. [7]	4.98	1.03
	McKinley et al. [6]	1.8	0.72

## Data Availability

The data used to support the findings of this study are included within the article.
